# Incidence and risk factors of Pulmonary Complications after Cardiopulmonary bypass

**DOI:** 10.12669/pjms.334.12846

**Published:** 2017

**Authors:** Anjum Naveed, Hammad Azam, Humayoun Ghulam Murtaza, Rana Altaf Ahmad, Mirza Ahmad Raza Baig

**Affiliations:** 1 Dr. Anjum Naveed, (FCPS). Assistant Professor of Pulmonology, CPE Institute of Cardiology, Multan, Pakistan; 2 Hammad Azam, (FCPS Surgery). Assistant Professor of Cardiac Surgery, Sheikh Zayed Medical College and Hospital, Rahim Yaar Khan, Pakistan; 3 Humayoun Ghulam Murtaza, (DTCD, FCPS). Senior Registrar Pulmonology, Nishtar Medical College/Hospital, Multan, Pakistan; 4 Rana Altaf Ahmad, (DA, FCPS, M. Sc. Pain Medicine). Professor of Anesthesia and Critical Care, Executive Director, CPE Institute of Cardiology, Multan, Pakistan; 5 Mirza Ahmad Raza Baig, (BSc. Hons. CPT). Clinical Perfusionist, CPE Institute of Cardiology, Multan, Pakistan

**Keywords:** Cardiopulmonary bypass, Coronary artery bypass grafting, Pulmonary complications, Valvular heart surgery

## Abstract

**Objective:**

To determine the frequency of post-operative pulmonary complications (PPCs) after cardio-pulmonary bypass and association of pre-operative and intraoperative risk factors with incidence of PPCs.

**Methods:**

This study was an observational analysis of five hundred and seventeen (517) patients who underwent cardiac surgery using cardiopulmonary bypass. Incidence of PPCs and risk factors of PPCs were noted. Logistic regression was applied to determine the association of pre-operative and intraoperative risk factors with incidence of PPCs.

**Results:**

Post-operative pulmonary complications occurred in 32 (6.2%) patients. Most common post-operative pulmonary complication was atelectasis that occurred in 20 (3.86%) patients, respiratory failure in 8 (1.54%) patients, pneumonia in 3 (0.58%) patients and acute respiratory distress syndrome in 1 (0.19%) patients. The main risk factor of PPCs were advance age ≥ 60 years [odds ratio 4.16 (1.99-8.67), p-value <0.001], prolonged CPB time > 120 minutes [odds ratio 3.62 (1.46-8.97) p-value 0.003], pre-op pulmonary hypertension [odds ratio 2.60 (1.18-5.73), p-value 0.016] and intraoperative phrenic nerve injury [odds ratio 7.06 (1.73-28.74), p-value 0.002]. Operative mortality was 9.4% in patients with PPCs and 1.0% in patients without PPCs (p-value 0.01).

**Conclusion:**

The incidence of post-operative pulmonary complications was 6.2% in this study. Advanced age (age ≥ 60 years), prolonged CPB time (CPB time > 120 minutes), pre-op pulmonary hypertension and intraoperative phrenic nerve injury are independent risk factors of PPCs after surgery.

## INTRODUCTION

Despite many advances in perioperative care, post-operative pulmonary complications (PPCs) still remain the leading cause of morbidity and death after adult cardiac surgery.^1^,^2^ PPCs are associated with increased length of hospital stay and these have a great influence on health care cost in cardiac surgery patients.^3^ Development of pulmonary complications after cardiopulmonary bypass is multi-factorial; alteration in the function of chest muscles and wall due to median sternotomy, systemic inflammatory response syndrome initiated by establishing cardiopulmonary bypass, phrenic nerve damage caused by administration of cold saline in the pericardial cavity during cardiac arrest and alveolar edema caused by left ventricular distension and elevated pressure in the pulmonary vasculature are considered the main contributing factors for this lethal complication.^4^ The reported frequency of pulmonary complications after cardiac surgery varies from 6% to 70% depending upon the criteria used to define pulmonary complications.^5^

Very few studies have focused on the intraoperative and postoperative risk factors responsible for the development of pulmonary complications in patients undergoing cardiac surgery using cardiopulmonary bypass.^6^,^7^ In present study we determined the frequency of post-operative pulmonary complications (PPCs) and inter-operative factors associated with PPCs in patients undergoing cardiac surgery using cardio-pulmonary bypass.

## METHODS

This prospective observational study was arranged in Ch. Pervaiz Elahi Institute of Cardiology Multan Pakistan and had approval from department of academic affairs of the hospital. A total number of 517 patients undergoing cardiac surgery using cardiopulmonary bypass (CPB) from Jan 2015 to August 2016 were included. Patients undergoing coronary artery bypass grafting (CABG) and valvular operations were selected. Patients undergoing congenital cardiac procedures were excluded.

In all patients standard procedures were used for surgery. All procedure was done through median skin incision. Standard cardiopulmonary bypass apparatus was used in all patients incorporating membrane oxygenator and arterial line filters. Venus cannulation was done through right atrium using either two single venous cannulas or a two stage single venous cannula. And arterial cannulation was done through ascending aorta using either angles tip or straight tip aortic perfusion cannula. Lactated ringer was used to prime CPB circuit. After establishing CPB, temperature was lowered to 30 to 28 degree Celsius to maintain moderate hypothermia during surgery. Cold blood cardioplegia was used to arrest and protect the heart of patients undergoing CABG and tepid blood cardioplegia was used for valvular procedures after application of aortic cross-clamp. All patients were weaned off from CPB after re-warming the patient to 36.5 to 37 degree Celsius. All patients were shifted to intensive care unit (ICU) in stable condition. And weaned off from mechanical ventilation in ICU.

The senior anesthetist and the pulmonologist of the hospital noted data regarding post-operative pulmonary complications. Duration of mechanical ventilation > 48 hours or need for re-intubation of patients was considered as respiratory failure. Pneumonia was labeled by the presence of fever and sputum along with presence of findings of pneumonia on chest X-rays and on laboratory reports. Any death during hospital stay or within one month after surgery due to the results of surgical complications was considered operative mortality.

Data analysis was done using SPSS v23 software. Chi-square tests were used to determine the effect of post-operative pulmonary complications on operative mortality and to compare the frequency of PPCs in valvular and CABG patients. Logistic regression analysis was used to determine the association of pre-operative and intra-operative variables on the incidence of PPCs after surgery. P-value ≤ 0.05 was taken as significant effect.

## RESULTS

**Fig. 1:**
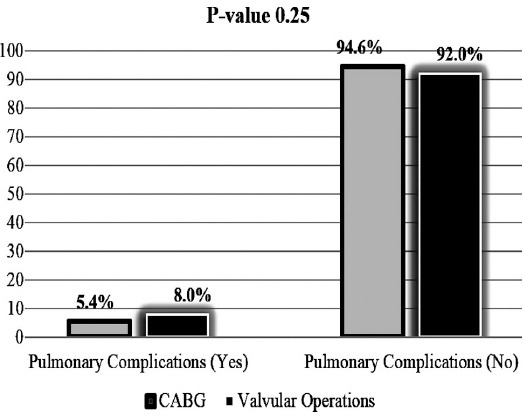
Association of Types of procedures with the incidence of post-operative pulmonary complications.

This study included five hundred and seventeen patients (517). The mean age of study participants was 49.00±15.36 years. There were 121 (23.4%) female patients. Out of these 517, 349 (67.5%) patients underwent coronary artery bypass grafting (CABG), 5 (1.0%) CABG + aortic valve replacement and 163 (31.5%) patients underwent valvular operations. Post-operative pulmonary complications occurred in 32 (6.2%) patients. Most common post-operative pulmonary complication was atelectasis which occurred in 20 (3.86%) patients, respiratory failure in 8 (1.54%) patients, pneumonia in three (0.58%) patients and acute respiratory distress syndrome in one (0.19%) patients (Table-I). There was no significant difference in the incidence of pulmonary complications between the patients who underwent CABG or valvular operations (Fig. 1).

**Table-I:** Type of operations and incidence of post-operative pulmonary complications.

*Variable*	*Value*

*Types of Procedures*	
CABG	349 (67.5%)
CABG plus Aortic valve replacement	5 (1.0%)
Valvular Operations	163 (31.5%)
a) Mitral Valve Replacement (%)	105 (20.3%)
b) Aortic Valve Replacement (%)	40 (7.7%)
c) Aortic plus Mitral Valve Replacement (%)	18 (3.5%)
Post-operative Pulmonary Complications	32 (6.2%)
a) Pneumonia	3 (0.58%
b) Respiratory Failure	8 (1.54%)
c) Acute respiratory distress syndrome	1 (0.19%)
d) Atelectasis	20 (3.86%)

There was significant difference in operative mortality in patients who developed post-operative pulmonary complications as compared to the patients in whom there was no incidence of pulmonary complications. Total operative mortality was 8 (1.54%), and out of these eight, three (9.4%) patients were those of having PPCs after cardiac surgery and remaining five (1.0%) were those without PPCs (p-value 0.01). Out of total three mortalities in PPCs patients, there was one patients who developed acute respiratory distress syndrome, and two in patients with respiratory failure.

In our study, the main risk factor of PPCs were advance age (age ≥ 60 years), prolonged CPB time CPB time > 120 minutes), pre-op pulmonary hypertension and intraoperative phrenic nerve injury (Table-III).

**Table-II:** Association of pulmonary complications with operative mortality.

*Operative Mortality*	*Pulmonary Complications (Yes)*	*Pulmonary Complications (No)*	*P-value*
Yes	3 (9.4%)	5 (1.0%)	0.01
No	29 (90.6%)	480 (99.0%)	

**Table-III:** Multivariate analysis of risk factors of post-operative pulmonary complications.

*Risk factors*	*OR*	*(95% CI)*	*p-value*
Age ≥ 60 years	4.16	1.99-8.67	<0.001
Pre-op Pulmonary hypertension	2.60	1.18-5.73	0.014
CPB time > 120 minutes	3.62	1.46-8.97	0.003
Phrenic nerve injury	7.06	1.73-28.74	0.002

## DISCUSSION

Pulmonary complications are rare after cardiopulmonary bypass but can be life threatening in some cases. The documented incidence of PPCs ranges from 3% to 16% after CABG and 5%-7% after valvular heart surgery.^2^,^8^-^10^ In our study, PPCs occurred in 6.2% patients after surgery, with 8.0% incidence in patients with valvular operations and 5.4% in patients with after CABG. The incidence of PPCs in our study was comparable to the results of other international studies.

In this study, atelectasis occurred in 20 (3.86%) patients. In the study of Al-Qubati et al. incidence of post-op atelectasis was 1.11%.^11^ Incidence of atelectasis in our study was comparable to this study. Other studies have reported 14.1% to 70% incidence of post-operative atelectasis after cardiac surgery.^12^,^13^ The reduction in incidence of atelectasis in our study may be due to the practice of pre-operative incentive spirometry practice in all patients before surgery and the use of positive end expiratory pressure (7-8 cmH_2_O) and CPAP at 5 cmH_2_O during mechanical ventilation after surgery.^12^,^14^,^15^

In our study, post-operative pneumonia occurred in 0.58% patients. The reported incidence of pneumonia after cardiac surgery is 2.0%-2.7%.^11^,^16^ Some studies on the other hand have reported high incidence of pneumonia in our study after surgery with the incidence rate ranging from 15%-20%.^17^ The incidence of pneumonia was less as compared to these studies.

Many studies have reported different risk factors of PPCs after cardiac surgery, these include; advanced age, smoking, chronic obstructive pulmonary disease (COPD), diabetes, obesity, gastrointestinal hemorrhage, post-surgery lung injury, damage to the phrenic nerve during surgery, sternal wound infections and re-exploration after surgery.^18^,^19^ Ji Q et al. concluded that older age, preoperative heart failure, preoperative PaO_2,_ prolonged CPB duration and phrenic nerve damage are the most common risk factors of PPCs after cardiac surgery.^7^ In the study of Al-Qubati et al. increased intensive care unit stay of patients, massive need of blood transfusions after surgery and re-exploration after surgery were the main risk factors of PPCs after surgery.^11^ In our study, the main risk factors of PPCs after CPB were age ≥ 60 years at the time of surgery, pre-op pulmonary hypertension, CPB time > 120 minutes and injury to the phrenic nerve during surgery. In this study, there was no significant effect of re-exploration on incidence of PPCs (odds ratio 1.24 (0.57-2.69 and p-value 0.58). Damage to the phrenic nerve during surgery was the strongest predictor of PPCs after surgery. In a previous study conducted in our institute in 2012, frequency of prolonged mechanical ventilation (respiratory failure) was 4.76%, and ARDS 0.08 and pneumothorax was 0.91%.^20^ In current study, there was no incidence of pneumothorax in any patient. Therefore, by comparing our results with that previous study, we can conclude that the incidence of PPCs have reduced in our hospital with the passage of time.

Cardiopulmonary bypass is considered a major risk factor of post-operative complications after cardiac surgery due to the activation of systemic inflammatory response syndrome (SIRS) associated with CPB. This results in activation of neutrophils and compliments systems and ultimately the production of oxygen free radicals.^21^,^22^ All these have deleterious effects on all organs of the body and lungs are more prone to damage due to SIRS resulting in development of PPCs, increased duration of mechanical ventilation and hence prolonged ICU stay of patients.^23^ Development of intra-operative strategies to reduce the SIRS and damage to the phrenic nerve during surgery can help to reduce the incidence of PPCs.

### Authors’ Contribution

**AN:** Conceived, designed the research work and accountable for the originality of the research work.

**HA:** did data collection and wrote the methods.

**HGA:** did analysis of data and writing of manuscript.

**MARB & RAA:** did review and give final approval for this research work.
